# Methotrexate, Doxorubicin, and Cisplatin Versus Methotrexate, Doxorubicin, and Cisplatin + Ifosfamide in Poor Responders to Preoperative Chemotherapy for Newly Diagnosed High-Grade Osteosarcoma (JCOG0905): A Multicenter, Open-Label, Randomized Trial

**DOI:** 10.1200/JCO-24-01281

**Published:** 2025-03-26

**Authors:** Hiroaki Hiraga, Ryunosuke Machida, Akira Kawai, Toshiyuki Kunisada, Tsukasa Yonemoto, Makoto Endo, Yoshihiro Nishida, Akihito Nagano, Keisuke Ae, Shinichirou Yoshida, Kunihiro Asanuma, Junya Toguchida, Taisuke Furuta, Robert Nakayama, Toshihiro Akisue, Toru Hiruma, Takeshi Morii, Hideki Nishimura, Koji Hiraoka, Masanobu Takeyama, Makoto Emori, Satoshi Tsukushi, Hiroshi Hatano, Hiroyuki Kawashima, Kazuo Isu, Kazuhiro Tanaka, Tomoko Kataoka, Haruhiko Fukuda, Yukihide Iwamoto, Toshifumi Ozaki

**Affiliations:** ^1^ Musculoskeletal Oncology, NHO Hokkaido Cancer Center, Sapporo, Japan; ^2^ Japan Clinical Oncology Group Data Center/Operations Office, National Cancer Center Hospital, Tokyo, Japan; ^3^ Musculoskeletal Oncology, National Cancer Center Hospital, Tokyo, Japan; ^4^ Orthopaedic Surgery, Faculty of Medicine, Okayama University, Okayama, Japan; ^5^ Orthopaedic Surgery, Chiba Cancer Center, Chiba, Japan; ^6^ Orthopaedic Surgery, Graduate School of Medical Sciences, Kyushu University, Fukuoka, Japan; ^7^ Department of Rehabilitation Medicine, Nagoya University Hospital, Nagoya, Japan; ^8^ Orthopaedic Surgery, Gifu University School of Medicine, Gifu, Japan; ^9^ Orthopaedic Surgery, Cancer Institute Hospital, Tokyo, Japan; ^10^ Orthopaedic Surgery, Tohoku University Graduate School of Medicine, Sendai, Japan; ^11^ Orthopaedic Surgery, Mie University, Tsu, Japan; ^12^ Department of Tissue Regeneration, Center for Induced Pluripotent Stem Cell Research and Application, Institute for Frontier Medical Sciences, Kyoto University, Kyoto, Japan; ^13^ Orthopaedic Surgery, Hiroshima University, Hiroshima, Japan; ^14^ Department of Orthopaedic Surgery, Keio University School of Medicine, Tokyo, Japan; ^15^ Orthopaedic Surgery, Kobe University, Kobe, Japan; ^16^ Bone and Soft Tissue Tumour Surgery, Kanagawa Cancer Center, Yokohama, Japan; ^17^ Orthopaedic Surgery, Kyorin University, Tokyo, Japan; ^18^ Orthopaedic Surgery, Kagawa University Hospital, Takamatsu, Japan; ^19^ Orthopaedic Surgery, Kurume University Hospital, Kurume, Japan; ^20^ Orthopaedic Surgery, Yokohama City University Hospital, Yokohama, Japan; ^21^ Orthopaedic Surgery, Sapporo Medical University Hospital, Sapporo, Japan; ^22^ Orthopaedic Surgery, Aichi Cancer Center, Nagoya, Japan; ^23^ Musculoskeletal Oncology, Niigata Cancer Center Hospital, Niigata, Japan; ^24^ Orthopaedic Surgery, Niigata University Medical & Dental Hospital, Niigata, Japan; ^25^ Orthopaedic Surgery, Higashi Sapporo Hospital, Sapporo, Japan; ^26^ Advanced Medical Sciences, Oita University Faculty of Medicine, Oita, Japan; ^27^ Orthopaedic Surgery, Kyushu Rosai Hospital, Kitakyusyu, Japan

## Abstract

**PURPOSE:**

Our previous NECO phase II studies on high-grade osteosarcoma suggested that administering ifosfamide (IF; 16 g/m^2^ [4g/m^2^ once on day 1, then 2g/m^2^ once on days 2-7] × six) to patients showing a poor response (PrRsp) to preoperative chemotherapy with methotrexate, doxorubicin, and cisplatin (MAP) improves their prognoses. In this Japan Clinical Oncology Group (JCOG) study, JCOG0905, we aimed to investigate the efficacy and safety of IF in patients with PrRsp.

**METHODS:**

JCOG0905 is a multicenter, open-label, multi-institutional, randomized trial. Eligible patients (50 years and younger) had resectable, high-grade osteosarcoma (stage II or III, Union for International Cancer Control TNM) of the extremities, limb girdles, and thoracic wall. After two MAP cycles and tumor resection, patients with PrRsp were randomly assigned to either the MAP or MAP plus 15 g/m^2^ (3g/m^2^ once daily on days 1-5) × six IF (MAP + IF [MAPIF]) group. The primary end point was disease-free survival (DFS); secondary end points were overall survival (OS) and safety. The planned sample size was 100 patients with a one-sided α of .1 and a power of 0.7, assuming a 3-year DFS of 50% and 65% for MAP and MAPIF, respectively. This trial is registered with the Japan Registry of Clinical Trials (jRCT; jRCTs031180126).

**RESULTS:**

Of the 287 patients registered between February 2010 and August 2020, 51 and 52 patients with PrRsp were assigned to the MAP and MAPIF groups, respectively. As of March 2022, DFS did not differ between groups (hazard ratio [HR], 1.05 [95% CI, 0.55 to 1.98]) and OS was numerically inferior in the MAPIF group (HR, 1.48 [95% CI, 0.68 to 3.22]). Nine and zero patients in the MAPIF and MAP groups discontinued treatment because of adverse events, respectively.

**CONCLUSION:**

Evidence from JCOG0905 does not support the addition of IF for patients with PrRsp.

## INTRODUCTION

High-grade osteosarcoma, which has a poor prognosis with tumor resection alone, has shown marked improvement with multidrug therapy.^1,2^ Over the past three decades, large randomized controlled trials (RCTs) have adopted standard treatment using three-drug combination therapy encompassing methotrexate, doxorubicin, and cisplatin (MAP) before and after tumor resection.^3-7^ Introduction of preoperative chemotherapy has made the histologic response of resected tumors a strong prognostic factor.^3,8-10^ Patients showing a good histologic response had a 5-year overall survival (OS) of 68%-84%, whereas those with a poor response (PrRsp) had a 5-year OS of 41%-49%.^11-13^ Whether adding a new drug to the poor responder population improves prognosis beyond additional adverse events remains unclear.^10,14-16^

**CONTEXT**

**Key Objective**
Does the postoperative addition of higher-dose ifosfamide (IF) without etoposide improve disease-free survival (DFS) in patients showing poor response after neoadjuvant chemotherapy with methotrexate, doxorubicin, and cisplatin (MAP)?
**Knowledge Generated**
Adding adjuvant IF (15 g/m^2^ [3 g/m^2^ once daily on days 1-5] × six courses) to reduced-dose MAP showed no improvement in DFS and resulted in numerically inferior overall survival compared with MAP alone. Higher IF doses caused central nervous system and severe hematologic toxicities, respiratory distress indicative of allergic reactions, and reduced protocol completion rates; however, no secondary malignancies have been reported to date.
**Relevance *(R.G. Maki)***
While the assessment of the response to neoadjuvant therapy is an attractive time to modify therapy, in the case of osteosarcoma, the addition of high dose IF did not improve upon the outcomes with MAP alone in primary osteosarcoma patients. These data are consistent with prior randomized trials using IF starting in the neoadjuvant setting, and continue to support MAP alone as one standard of care for neoadjuvant/adjuvant therapy for primary osteosarcoma.*
*Relevance section written by *JCO* Associate Editor Robert G. Maki, MD, PhD, FACP, FASCO.

EURAMOS-1, the first RCT to address this issue, showed that adding a cumulative total dose of ifosfamide (IF) at 60 g/m^2^ (2.8 g/m^2^ once daily on days 1-5 × 3 and 1.8 g/m^2^ once daily on days 1-5 × 2) and 1.5 g/m^2^ etoposide (100 mg/m^2^ once daily on days 1-5 × 3 courses) to postoperative MAP therapy for poor responders after preoperative MAP therapy did not improve prognosis or increase adverse events.^4,5^

By contrast, the NECO93J and 95J clinical trials in Japan suggested that adding a higher dose of IF alone to postoperative MAP therapy for poor responders might improve prognosis.^14^ Therefore, the Japan Clinical Oncology Group (JCOG) JCOG0905 conducted a trial to confirm whether the addition of a cumulative total dose of IF at 90 g/m^2^ (3 g/m^2^ once daily on days 1-5 × six) to poor responders would improve their prognosis, outweighing the disadvantages of additional adverse events and extended treatment duration.

## METHODS

### Study Design and Participants

JCOG0905 is a multicenter, open-label, randomized trial conducted in Japan. Eligibility criteria for first registration included patients 40 years and younger with resectable histologically proven high-grade extremity or limb girdle osteosarcoma (stage IIA, IIB, III, Union for International Cancer Control [UICC] TNM sixth edition) not extending to the sacrum and an Eastern Cooperative Oncology Group performance status of 0 or 1. The age limit was increased to 50 years, and tumors in the clavicle, sternum, and ribs were included to boost enrollment after protocol revision.

Eligibility for the second registration required two preoperative MAP cycles, complete resection of the primary tumor, one postoperative doxorubicin and cisplatin cycle without disease progression, and a known histologic response.

The trial started with 26 institutions of the JCOG Bone and Soft Tissue Tumor Study Group and increased to 37 to expedite enrollment. Each institution obtained ethical approval from their Ethics Review Boards before enrollment. All trial participants or their parents provided written informed consent. With the enactment of the 2018 Clinical Research Act, JCOG0905 became a specified clinical trial, reviewed by the Certified Review Board of the National Cancer Center Hospital.

### Random Assignment and Masking

At the time of the second registration, patients with PrRsp were randomly assigned to the MAP arm (received MAP therapy) or MAPIF arm (received MAP + IF, MAPIF therapy) using a minimization method with biased coin assignment balanced on T factors (T1 *v* T2 and T3): classification by tumor size following the American Joint Committee on Cancer (AJCC)/UICC sixth edition, tumor location (lower limb girdle *v* other bones), and institution. Patients and the treating physicians were not blinded to either the treatment or outcome assessment. Patients with good response (good responders) received MAP therapy without random assignment.

### Procedures

Registered patients received two cycles of preoperative MAP therapy, comprising cisplatin, doxorubicin, and high-dose methotrexate (Table 1).

**TABLE 1.** Prescribed Dosages

Drug Abbreviation	Drug Name	Dose (mg/m^2^/d)	Administration Route/Time	Administration Schedule
AP	Doxorubicin	30	IV/24 hours	Once daily on days 1 and 2
	Cisplatin	120 (≤29 years old)	IV/24 hours	Once on day 1
		100 (≥30 years old)		
A	Doxorubicin	30 (≤39 years old)	IV/24 hours	Once daily on days 1, 2, and 3
		25 (≥40 years old)		
M	Methotrexate	12,000 (≤19 years old)	IV/4-6 hours	Once on day 1
		10,000 (≥20 years old)		
		8,000 (≥40 years old)		
IF	Ifosfamide	3,000 (≤40 years old)	IV/6 hours	Once daily on days 1, 2, 3, 4, and 5
		2,400 (≥40 years old)		

Abbreviation: IV, intravenous.

Tumor excision followed within 5 weeks of completing preoperative chemotherapy. Histologic responses were evaluated at each institute and graded as follows: grades 1 and 2 represented poor responders and grades 3 and 4 indicated good responders (Appendix Table A1, online only).^10,17^

Postsurgery, all patients received one cycle of doxorubicin and cisplatin before second registration to prevent delays in postoperative chemotherapy.

After the second registration, the good responders and MAP arm continued MAP therapy. The MAPIF arm was started on MAPIF therapy including six courses of IF and MAP with doxorubicin reduced to 57% and methotrexate to 80% to balance adverse effects and treatment duration between the two arms.

**FIG A1.**
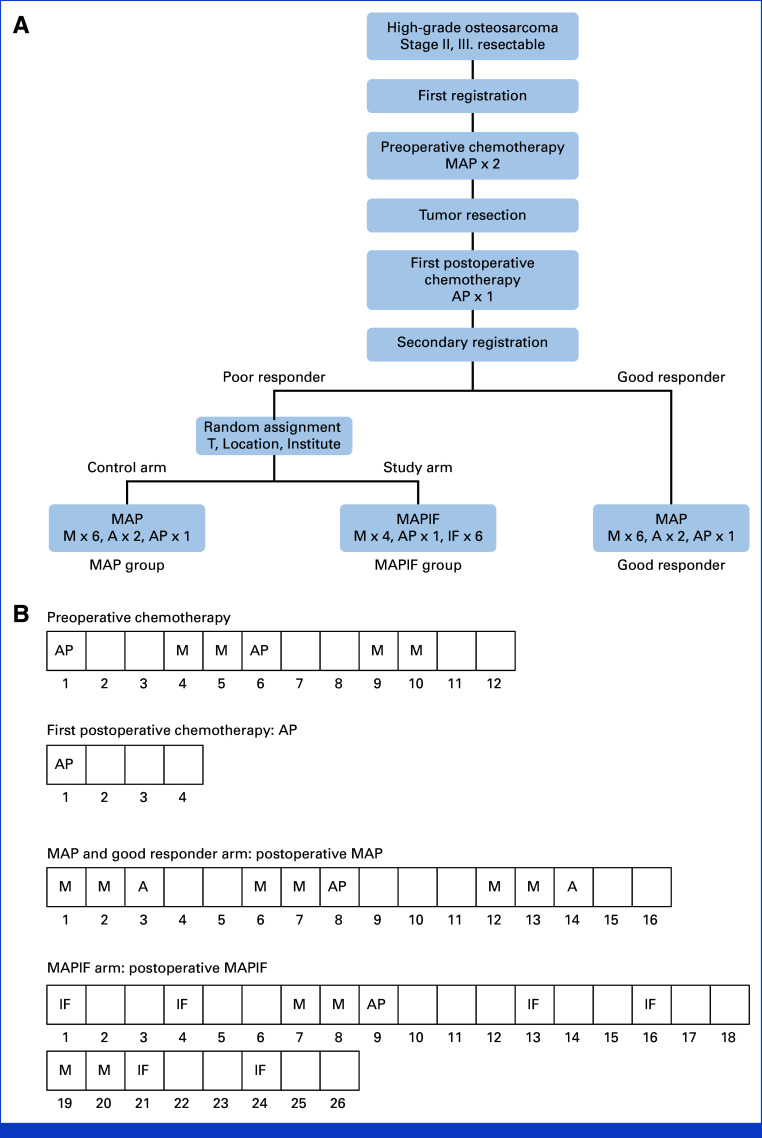
(A) Scheme of JCOG0905. (B) Scheme of each treatment segment. Registered patients received two cycles of preoperative MAP therapy: AP was administered at weeks 1 and 6, and high-dose M at weeks 4, 5, 9, and 10. Two additional M treatments were permitted if fabricating and delivering an expandable prosthesis for skeletally immature children were time-consuming. Wide excision of the tumor was performed within 5 weeks of starting the last course of preoperative chemotherapy. All patients received one cycle of AP between 1 and 5 weeks after surgery. After the second registration, the subsequent treatment was to be started within 1 week. Good and poor responders randomly assigned to the MAP arm were administered M at weeks 1, 2, 6, 7, 12, and 13; A at weeks 3 and 14; and AP at week 8. Poor responders randomly assigned to the MAPIF arm were administered IF at weeks 1, 4, 13, 16, 21, and 24; M at weeks 7, 8, 19, and 20; and AP at week 9. A, doxorubicin; AP, doxorubicin and cisplatin; IF, ifosfamide; JCOG, Japan Clinical Oncology Group; M, methotrexate; MAP, methotrexate, doxorubicin, and cisplatin; MAPIF, MAP + ifosfamide; T, T factor.

Dexrazoxane is not available for myocardial protection in Japan. The study design, treatment schedule, and drug doses are described in the Appendix (Figs A1A and A1B).

### Outcomes

The primary end point was disease-free survival (DFS) for poor responders, defined as the time from the second registration to the first event, including relapse, secondary malignancy, or death because of any cause. Secondary end points included DFS for good responders, OS in each arm from the second registration, proportion of patients progressing before surgery, and toxicity. OS was defined as the time from second registration until death from any cause. Data were collected centrally by the JCOG Data Center, alongside toxicity assessment following Common Terminology Criteria for Adverse Events version 3.0 at each institute.

### Statistical Analysis

Assuming a 3-year DFS of 50% in the MAP arm and 65% in the MAPIF arm, 92 patients and 58 events for the MAP and MAPIF arms were required to achieve the desired power of 70%, according to Schoenfeld and Richter's method,^18^ with an accrual period of 6 years, a 3-year follow-up, and a one-sided α of .1. The sample size was set to 200 patients at the first registration and 100 (50 patients in each arm) at the second registration, assuming that approximately 50% of patients at the first registration would be poor responders, followed by random assignment.

Two interim analyses were conducted at the JCOG Data Center from the perspective of both futility and efficacy. The first, which was held after the data of half of the planned randomly assigned patients were obtained, assessed the appropriateness of continuing accrual, whereas the second evaluated whether follow-up should continue after completing the second registration. The second analysis was conducted after complete enrollment for all randomly assigned patients in the second registration. The multiplicity of testing in the interim and primary analyses was adjusted using the Lan and DeMets α spending function with O'Brien and Fleming types to maintain a 10% overall α error. The trial was to be terminated at the interim analyses if MAPIF showed statistically significant superiority to MAP in DFS. By contrast, if the DFS curve of the MAPIF arm was lower than MAP's, trial termination was considered, regardless of statistical results.

In this second analysis, DFS between the MAP and MAPIF arms was compared using a stratified one-sided log-rank test, with T factors (T1 *v* T2 and T3) as a stratification factor in the intention-to-treat population. The primary end point results are reported using a one-sided *P* value on the basis of trial design. In addition, we report two-sided *P* values as a reference per *JCO* guidelines. Hazard ratios (HRs) and their 95% CIs were estimated using Cox regression stratified according to T factors for DFS and unstratified Cox regression for OS. DFS and OS were estimated using the Kaplan-Meier method. The CI for the proportion of patients progressing before surgery was estimated using the Clopper-Pearson method. Median follow-up among all poor responders was calculated using the reverse Kaplan-Meier method, which reverses the status indicator of DFS events and censoring. All statistical analyses were performed using SAS version 9.4 (SAS Institute, Cary, NC) software. This trial was registered with Japan Registry of Clinical Trials (jRCT; jRCTs031180126).

### Role of the Funding Source

The funders had no role in the study design; data collection, analysis, and interpretation; report writing; or publication decision.

## RESULTS

### Patients

**FIG 1.**
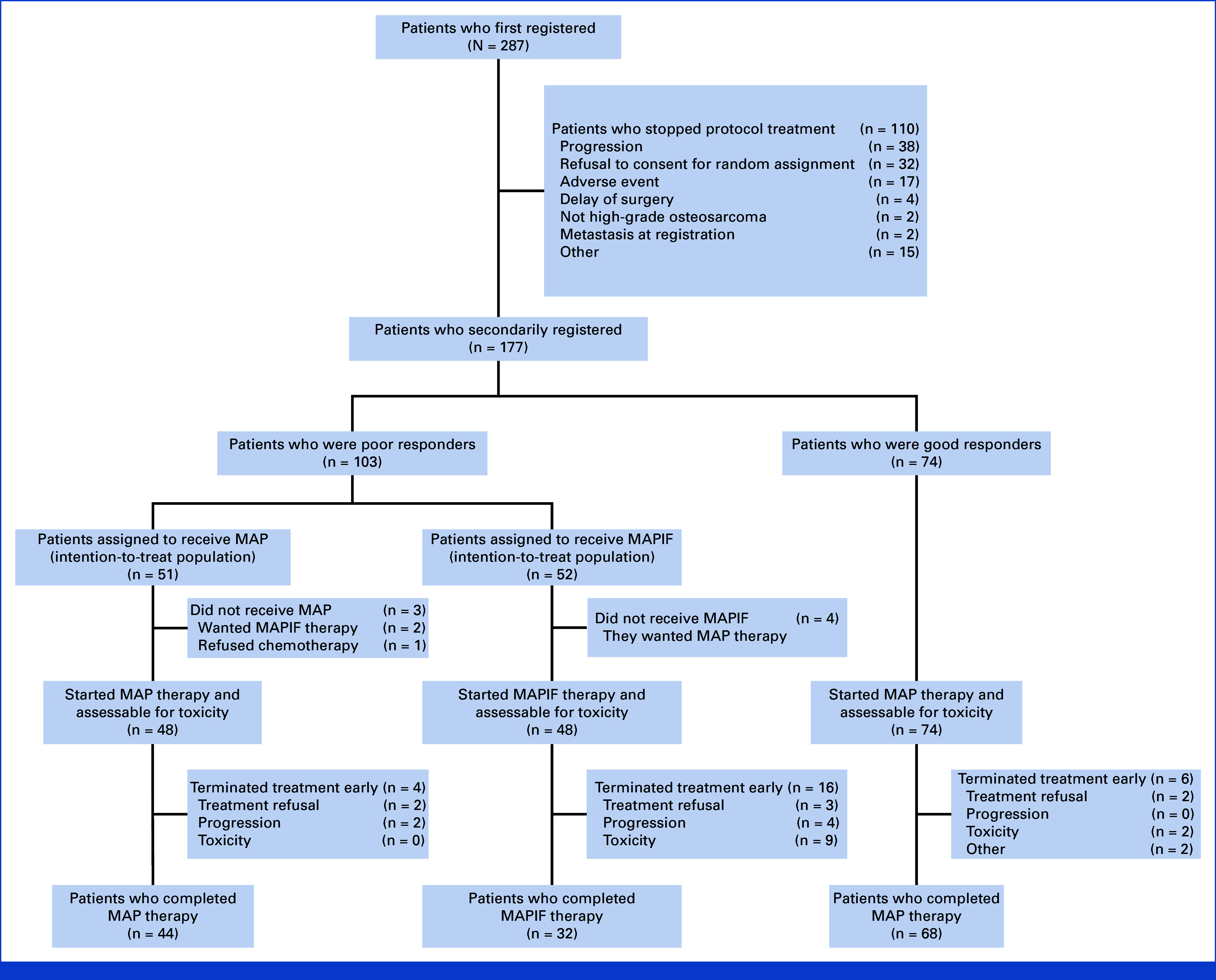
CONSORT diagram. MAP, methotrexate, doxorubicin, and cisplatin; MAPIF, MAP + ifosfamide.

Between February 16, 2010, and August 21, 2020, 287 patients were registered until 100 poor responders were randomly assigned. The second registration closed on January 22, 2021, with 103 poor responders assigned randomly: 51 and 52 to receive MAP and MAPIF therapy, respectively (Fig 1). Of the 287 patients, 18 were deemed ineligible, including three whose diagnosis was changed and did not meet the criteria, 11 with insufficient hepatitis B testing, one with lung metastasis postregistration, and one who did not meet registration regulations. Two patients started treatment before registration, violating the registration protocol. Excluding three patients whose diagnoses were changed, 11 of the remaining 15 patients proceeded to secondary registration. The most common reasons for not proceeding to the second registration were disease progression and patient refusal of random assignment.

The median follow-up was 67.1 months (95% CI, 54.9 to 79.2 months) for all poor responders. Three patients in the MAP arm were lost during follow-up at 1.6, 2.5, and 4.1 years postrandomization. On the basis of the second interim analysis results, JCOG's Data and Safety Monitoring Committee recommended study termination as the HR for the primary end point, DFS, exceeded the prespecified value of 1.0.

Baseline characteristics at the first registration of randomly assigned patients were well-balanced between both arms (Table 2).

**TABLE 2.** Patient Characteristics at First Registration

Characteristic	MAP (n = 51)	MAPIF (n = 52)	Good Responder (n = 74)
Sex, No. (%)			
Male	32 (63)	35 (67)	44 (59)
Female	19 (37)	17 (33)	30 (41)
Age at first registration, years			
Median (IQR)	15 (13-19)	15 (13-19)	14 (11-16)
Range	8-36	9-40	4-28
Site of tumor, No. (%)			
Humerus proximal	2 (4)	3 (6)	10 (14)
Radius diaphysis	0	0	1 (1)
Radius distal	0	0	1 (1)
Femur proximal	0	1 (2)	3 (4)
Diaphysis	4 (8)	1 (2)	1 (1)
Distal	26 (51)	26 (50)	37 (50)
Tibia proximal	14 (27)	17 (33)	15 (20)
Diaphysis	1 (2)	1 (2)	0
Distal	2 (4)	1 (2)	2 (3)
Fibula proximal	1 (2)	2 (4)	3 (4)
Distal	1 (2)	0	0
Calcaneus	0	0	1 (1)
Location of tumor, No. (%)			
Upper limb girdle	0	0	0
Lower limb girdle	0	0	0
Sternum, rib	0	0	0
Upper limb	2 (4)	3 (6)	12 (16)
Lower limb	49 (96)	49 (94)	62 (84)
T factor, No. (%)			
T1	17 (33)	18 (35)	22 (30)
T2	32 (63)	31 (60)	51 (69)
T3	2 (4)	3 (6)	1 (1)
Pathologic fracture at diagnosis, No. (%)			
No	48 (94)	46 (88)	66 (89)
Yes	3 (6)	6 (12)	8 (11)
Histologic subtype, No. (%)			
Conventional	51 (100)	51 (98)	70 (95)
Telangiectatic	0	0	3 (4)
Round cell	0	0	1 (1)
High-grade surface	0	1 (2)	0
Histologic response, No. (%)			
Grade 1	22 (43)	30 (58)	0
Grade 2	29 (57)	22 (42)	0
Grade 3	0	0	54 (73)
Grade 4	0	0	20 (27)

Abbreviations: MAP, methotrexate, doxorubicin, and cisplatin; MAPIF, MAP + ifosfamide.

### Efficacy

**FIG 2.**
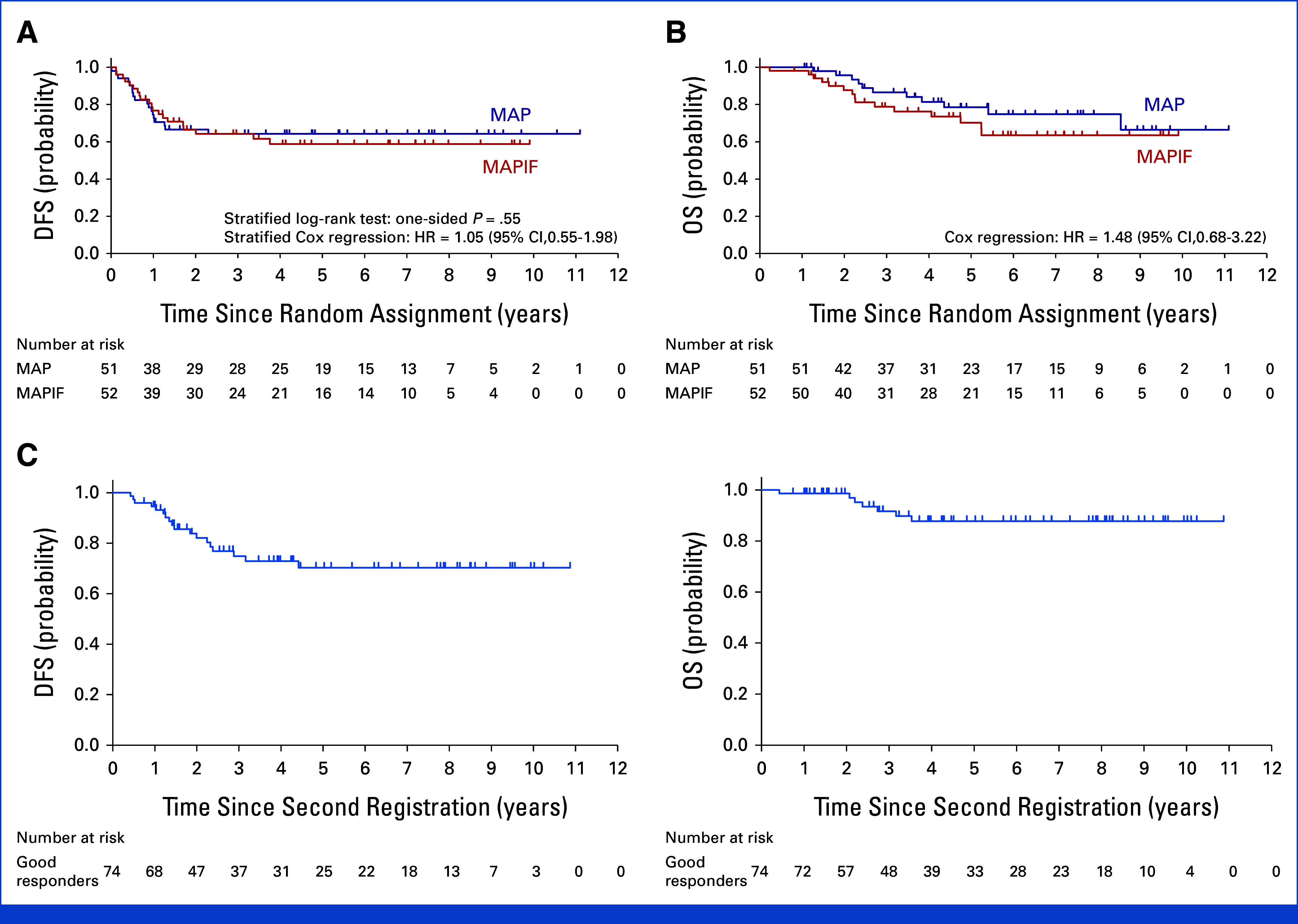
Survival analysis of the MAP and MAPIF arms. Kaplan-Meier curves showing (A) DFS and (B) OS for the MAP and MAPIF arms. (C) Kaplan-Meier curve showing DFS and OS in the good responders. DFS, disease-free survival; HR, hazard ratio; MAP, methotrexate, doxorubicin, and cisplatin; MAPIF, MAP + ifosfamide; OS, overall survival.

Among the 103 randomly assigned patients, 38 DFS events occurred (18 in the MAP arm, 20 in the MAPIF arm). The HR for DFS was 1.05 (95% CI, 0.55 to 1.98), with the 3-year DFS of 64.3% for both arms (stratified log-rank test: one-sided *P* = .55 and two-sided *P* = .89; Fig 2A).

There were 26 deaths (11 in the MAP arm, 15 in the MAPIF arm), all because of primary disease. The HR for OS was 1.48 (95% CI, 0.68 to 3.22), with 3-year OSs of 86.5% (95% CI, 72.4 to 93.7) and 78.8% (95% CI, 64.1 to 88.0) in the MAP and MAPIF arms, respectively (Fig 2B). Subgroup analyses for sex, T-factor, and histologic response showed no interactions with treatment effects for DFS or OS.

In the good responders, the 3-year DFS was 74.9% (95% CI, 62.1 to 83.9) and the 3-year OS was 91.7% (95% CI, 81.0 to 96.5; Fig 2C). There were seven deaths, five because of primary disease and two from treatment-related causes. The HRs for DFS and OS between good and poor responders were 0.59 (95% CI, 0.34 to 1.03) and 0.38 (95% CI, 0.17 to 0.88), respectively.

Among the 267 patients who underwent preoperative chemotherapy, 35 (13.1%) progressed before surgery (95% CI, 9.3 to 17.8).

The median cumulative total IF dose in the MAPIF arm was 79.8 g/m^2^ (IQR, 45.4-86.2) for 36 patients 19 years and younger, 77.1 g/m^2^ (IQR, 44.0-79.5) for nine patients age 20-29 years, and 80.7 g/m^2^ (IQR, 73.2-88.3) for two patients age 30-39 years (Table 3).

**TABLE 3.** Dose of Chemotherapeutic Agents Administered

CTX Agent	Groups	≤19 Years Old			20-29 Years Old			30-39 Years Old			40-50 Years Old		
		MAP	MAPIF	GR	MAP	MAPIF	GR	MAP	MAPIF	GR	MAP	MAPIF	GR
DOX	No.	38	30	68	9	7	6	1	2	0	0	1	0
	PCD, mg/m^2^	240	60	240	240	60	240	240	60	240	210	60	210
	CDAA, mg/m^2^												
	Median (IQR)	235.7 (228.7-240.9)	59.3 (56.8-61.2)	235.4 (226.3-241.4)	239.6 (237.8-245.2)	59.2 (51.7-60.3)	222.7 (141.3-234.3)	217	53.5 (48.2-58.9)			61.0	
	Mean (SD)	224.9 (41.9)	58.9 (3.8)	230.6 (26.1)	223.5 (51.3)	57.0 (5.2)	192.1 (59.6)		53.5 (7.5)				
	Range	83.5-253.8	48.0-64.7	90.3-271.3	88.5-253.1	47.8-62.3	94.7-237.0		48.2-58.9				
CDDP	No.	35	30	67	8	7	5	1	2	0	0	1	0
	PCD, mg/m^2^	120	120	120	120	120	120	100	100	100	100	100	100
	CDAA, mg/m^2^												
	Median (IQR)	118.4 (115.5-122.2)	118.8 (113.7-122.8)	119.0 (115.4-121.4)	121.1 (117.5-124.1)	119.6 (117.1-124.2)	114.3 (108.9-114.6)	91.6	98.5 (98.1-98.9)			101.7	
	Mean (SD)	117.0 (9.7)	116.6 (10.6)	117.6 (9.6)	120.7 (5.4)	115.9 (12.8)	111.0 (7.6)		98.5 (0.6)				
	Range	84.8-128.0	86.3-129.4	82.6-136.8	111.7-128.5	87.7-124.6	98.7-118.3		98.1-98.9				
MTX	No.	38	30	68	9	7	6	1	2	0	0	1	0
	PCD, mg/m^2^	72	48	72	60	40	60	60	40	60	48	32	48
	CDAA, mg/m^2^												
	Median (IQR)	70.5 (67.5-72.2)	46.6 (41.1-48.2)	70.8 (67.0-72.3)	60.5 (57.8-61.1)	39.6 (39.5-42.2)	47.6 (38.4-57.0)	55.6	39.6 (39.3-39.8)			40.8	
	Mean (SD)	66.5 (12.2)	42.1 (1.0)	67.8 (93.6)	55.7 (13.7)	40.4 (13.6)	45.3 (14.0)		39.6 (0.0)				
	Range	22.3-76.3	12.4-51.3	24.8-80.9	19.6-63.2	39.2-42.5	21.4-59.9		39.3-39.8				
IF	No.		36			9			2			1	
	PCD (g/m^2^)		90			90			90			72	
	CDAA, mg/m^2^												
	Median (IQR)		79.8 (45.4-86.2)			77.1 (44.0-79.5)			80.7 (73.2-88.3)			77.5	
	Mean (SD)		66.1 (26.9)			64 (28.5)			80.7 (10.7)				
	Range		28.0-97.0			14.6-95.0			73.2-88.3				

NOTE. All doses are cumulative total doses.

Abbreviations: CDAA, cumulative dose actually administered; CDDP, cisplatin; DOX, doxorubicin; GR, good responder; MAP, methotrexate, doxorubicin, and cisplatin; MAPIF, MAP + ifosfamide; MTX, methotrexate; PCD, prescribed cumulative dose; SD, standard deviation.

### Toxicity

Adverse event data from preoperative and first postoperative chemotherapy before the second registration are described in Appendix Table A2. One patient died during preoperative chemotherapy from septic shock after becoming pregnant and starting treatment after a stillbirth.

During postoperative chemotherapy, severe myelosuppression and febrile neutropenia (>30%) were observed in both arms. Toxicity data were available for all patients who started the allocated treatment (Table 4). Central and peripheral neuropathy and cystitis occurred in the MAPIF arm, with a high incidence of renal dysfunction as a late adverse effect. Two (2.7%) of 74 good responders had left ventricular dysfunction and died of heart failure after treatment; no such events occurred in the MAP or MAPIF arms.

**TABLE 4.** Postoperative Treatment-Related Adverse Events After Second Registration

Adverse Event	MAP^a^			MAPIF^a^			Good Responder			
	Grade 1 + 2	Grade 3	Grade 4	Grade 1 + 2	Grade 3	Grade 4	Grade 1 + 2	Grade 3	Grade 4	Grade 5
Toxicities routinely observed in adjuvant chemotherapy, No. (%)										
Leukocytes	3 (6)	20 (42)	25 (52)	1 (2)	1 (2)	46 (96)	2 (3)	29 (39)	42 (57)	0
Hemoglobin	21 (44)	22 (46)	5 (10)	12 (25)	21 (44)	15 (31)	18 (24)	38 (51)	18 (24)	0
Platelets	13 (27)	11 (23)	23 (48)	11 (23)	10 (21)	27 (56)	12 (16)	23 (31)	38 (51)	0
Neutrophils	1 (2)	0	47 (98)	1 (2)	1 (2)	46 (96)	2 (3)	2 (3)	69 (93)	0
Nonhematologic event	—	23 (48)	20 (42)	—	24 (50)	17 (35)	—	39 (53)	29 (39)	0
Nonhematologic event, except for AST or ALT	—	29 (60)	3 (6)	—	25 (52)	4 (8)	—	39 (53)	4 (5)	0
Bilirubin	14 (29)	1 (2)	0	15 (31)	0	0	19 (26)	0	0	0
AST	20 (42)	19 (40)	8 (17)	25 (52)	14 (29)	8 (17)	24 (32)	32 (43)	18 (24)	0
ALT	17 (35)	15 (31)	16 (33)	15 (31)	18 (38)	14 (29)	15 (20)	36 (49)	23 (31)	0
Creatinine	12 (25)	0	0	17 (35)	0	0	24 (32)	0	0	0
Hypokalemia	37 (77)	8 (17)	0	29 (60)	14 (29)	4 (8)	51 (69)	12 (16)	3 (4)	0
Hypomagnesemia^b^	27 (64)	1 (2)	3 (7)	26 (67)	2 (5)	2 (5)	50 (74)	5 (7)	0	0
Hearing	10 (21)	3 (6)	0	9 (19)	2 (4)	0	21 (28)	0	0	0
Left ventricular diastolic dysfunction	0	0	0	0	0	0	1 (1)	0	0	0
Left ventricular systolic dysfunction	1 (2)	0	0	0	0	0	1 (1)	0	0	0
Fever	9 (19)	0	0	8 (17)	2 (4)	0	10 (14)	5 (7)	0	0
Anorexia	33 (69)	4 (8)	0	38 (79)	4 (8)	0	54 (73)	3 (4)	0	0
Vomiting	20 (42)	0	0	25 (52)	2 (4)	0	31 (42)	1 (1)	0	0
Diarrhea	5 (10)	0	0	5 (10)	0	0	12 (16)	2 (3)	0	0
Hemorrhage, GU bladder	0	0	0	8 (17)	0	0	0	0	0	0
Febrile neutropenia	0	17 (35)	0	0	16 (33)	0	0	29 (39)	0	0
Confusion	0	0	0	0	0	0	1 (1)	0	0	0
Somnolence	2 (4)	0	0	1 (2)	1 (2)	0	1 (1)	0	0	0
Psychosis	0	0	0	1 (2)	0	0	0	0	0	0
Seizure	0	0	0	0	0	0	1 (1)	0	0	0
Encephalopathy	0	0	0	2 (4)	1 (2)	0	0	0	0	0
Involuntary movement	0	0	0	1 (2)	1 (2)	0	1 (1)	0	0	0
Neuropathy—motor	1 (2)	1 (2)	0	3 (6)	2 (4)	0	1 (1)	0	0	0
Neuropathy—sensory	1 (2)	1 (2)	0	8 (17)	1 (2)	0	5 (7)	0	0	0
Dizziness	1 (2)	0	0	1 (2)	0	0	0	0	0	0
Cystitis	0	0	0	11 (23)	2 (4)	0	1 (1)	0	0	0
Arrhythmia—supraventricular, ventricular	1 (2)	0	0	0	0	0	1 (1)	0	0	0
Mucositis—oral cavity, anus	17 (35)	3 (6)	0	11 (23)	1 (2)	0	39 (53)	1 (1)	0	0
Infection with grade 3 or 4 ANC^c^	0	1 (2)	0	0	0	0	0	2 (3)	0	0
Infection with grade 0-2 ANC^c^	0	0	0	0	0	0	0	2 (3)	0	0
Mood alteration—select: agitation, depression	0	0	0	1 (2)	0	0	2 (3)	0	0	0
Toxicities routinely observed after treatment, No. (%)										
Arrhythmia—supraventricular, ventricular	0	0	0	0	0	0	0	0	0	0
Left ventricular diastolic dysfunction	0	0	0	0	0	0	0	0	0	0
Left ventricular systolic dysfunction	0	0	0	0	0	0	1 (1)	0	0	2 (3)
Creatinine	4 (9)	0	0	15 (33)	0	0	8 (12)	0	0	0
Urinary electrolyte wasting	0	0	0	4 (10)	0	0	0	0	0	0
Toxicities not routinely observed in adjuvant chemotherapy and after treatment, No. (%)										
Wound complication, noninfectious		1			1		1			
Dyspnea					1^d^					
Infection with grade 3 or 4 ANC					3^e^			1		
Infection with grade 0-2 ANC		1			1			2		
Infection with unknown ANC		1					2	1		

NOTE. The proportion of each adverse event is calculated on the basis of the number of patients reporting each type of toxicity. Electrolyte abnormalities with an incidence of grade 3 or higher in <10% of the patients in any group were not noted.

Abbreviations: ANC, absolute neutrophil count; GU, genitourinary; MAP, methotrexate, doxorubicin, and cisplatin; MAPIF, MAP + ifosfamide.

^a^ Grade 5 adverse events did not occur in the MAP arm and MAPIF arm.

^b^ Collected after the first protocol revision.

^c^ Infection of the bronchus and lung.

^d^ Dyspnea accompanied by wheezing that occurred immediately after administration of IF, which was suspected to be an allergic reaction.

^e^ Two patients with herpes zoster, one with multiple liver abscesses.

In the MAP arm, two patients developed metastasis and two declined treatment because of stomatitis and hearing impairment; thus, 44 patients completed the treatment. In the MAPIF arm, four patients discontinued treatment after the first postoperative IF course: one each because of grade 3 somnolence and involuntary movement, respiratory distress, renal dysfunction, and hematologic toxicity. After the second IF course, four patients discontinued treatment: two because of disease progression, one with grade 3 encephalopathy, and one with grade 2 encephalopathy. In addition, eight patients discontinued treatment between the 5th and 10th courses: four because of hematologic toxicity, two because of disease progression, and two because of adjustment disorder and focusing on studies; finally, 32 patients completed the treatment.

Of the 35 patients who experienced progression before secondary registration, two developed secondary malignancy. One patient developed leukemia, and the other myelodysplastic syndrome; both died after bone marrow transplantation.

## DISCUSSION

Standard osteosarcoma treatment comprises preoperative MAP chemotherapy, tumor-wide resection, and postoperative chemotherapy.^4,5,8^ This approach allows for assessing histologic responses, a strong prognostic factor.^3,8-10^ Attempts have been made to improve prognosis by administering additional drugs to poor responders.^14-17,19^ In the EURAMOS-1 study, adding cumulative total doses of IF at 60 g/m^2^ and etoposide at 1.5 g/m^2^ to poor responders was investigated; however, the superiority of the experimental treatment was not confirmed.^5^ In this study, JCOG0905, adding a cumulative total dose of IF at 90 g/m^2^ (3 g/m^2^ once daily on days 1-5 × 6) for poor responders showed no improvement in prognoses. Adjuvant MAPIF did not have a stronger effect than MAP on DFS (HR, 1.05), and OS tended to be lower (HR, 1.48).

The therapeutic frameworks of the EURAMOS-1 and JCOG0905 trials for poor responders were similar, with partially overlapping accrual periods. When EURAMOS-1 yielded negative results, we considered whether to continue JCOG0905. Notwithstanding, we proceeded owing to the following differences: (1) JCOG0905 used a 1.5-fold higher IF dose, suggesting potential dose-dependent effects; (2) IF was administered earlier and more intensely, with reduced doxorubicin and methotrexate doses for MAP-resistant tumors; (3) etoposide was excluded because of unproven efficacy as a single-agent therapy and unclear benefits when combined with IF; and (4) EURAMOS-1 mainly enrolled European and American patients, whereas JCOG0905 enrolled patients exclusively in Japan, where no phase III osteosarcoma trials have been conducted. However, the results of both trials were almost identical in terms of the primary end point.

Among all patients registered in JCOG0905, two secondary malignancies were reported, both during the preoperative treatment period, leading to treatment discontinuation. No secondary malignancies were reported among patients who underwent second registration. In EURAMOS-1, three patients with secondary malignancies were reported among 301 patients in the MAP arm, as well as 10 patients among 298 patients in the MAP plus ifosfamide and etoposide (MAPIE) arm, indicating a potential association between the alkylating agent IF and etoposide.^5^ By contrast, in JCOG0905, a higher dose of IF without etoposide was administered. No secondary malignancies developed in patients after secondary registration in JCOG0905, possibly because of the short follow-up period (67.1 months [95% CI, 54.9 to 79.2 months]) and immature data. Therefore, monitoring for secondary malignancies is crucial during the long-term follow-up of JCOG0905.

In this trial, DFS between the MAP and MAPIF arms was comparable, but observed OS was shorter in the MAPIF arm although this difference was not statistically significant (HR, 1.476 [95% CI, 0.677 to 3.216]). This may be attributable to the lower cumulative doses of doxorubicin and methotrexate in the MAPIF arm (cumulative total doses of 240 mg/m^2^ and 96 g/m^2^, respectively) compared with the MAP arm (cumulative total doses of 420 mg/m^2^ and 120 g/m^2^). By contrast, in the EURAMOS-1 trial, the same doses were used in both the MAP and MAPIE (MAP + IF + VP-16) arms, resulting in similar DFS and OS. No trials have specifically examined the effects of different cumulative doxorubicin or methotrexate doses in a homogeneous patient population,^10^ making definitive interpretations challenging. However, the results may influence future clinical trials.

The difference in PrRsp rates between this trial (58%) and EURAMOS-1 (48%)^20^ is due to several factors: EURAMOS-1 used a higher cumulative dose of doxorubicin before operation; the evaluation methods might have differed, and the criteria for aggregating data differed, with JCOG0905 aggregating only data of patients who were secondarily registered, complicating direct comparison.

In the MAP arm, 44 (92%) patients completed treatment, whereas in the MAPIF arm, 32 patients (67%) completed treatment. The treatment completion proportion in a previous study, NECO95J, was 68.3%.^14^ Therefore, the current results were not notably lower; however, several early discontinuations owing to adverse reactions to IF after random assignment were unexpected. During the initial IF course, four patients discontinued treatment: one because of renal toxicity; one because of hematologic toxicity; one because of respiratory distress, considered an allergic reaction; and one because of grade 3 somnolence and involuntary movement considered a central nervous system impairment because of IF. In the second course of postoperative chemotherapy, one patient discontinued treatment because of grade 3 encephalopathy and one because of grade 2 encephalopathy. These encephalopathies were considered to be due to IF. Previous phase II trials have reported strong toxicity, including treatment-related deaths,^21-25^ confirming the significant toxicity associated with high-dose IF therapy.

**FIG A2.**
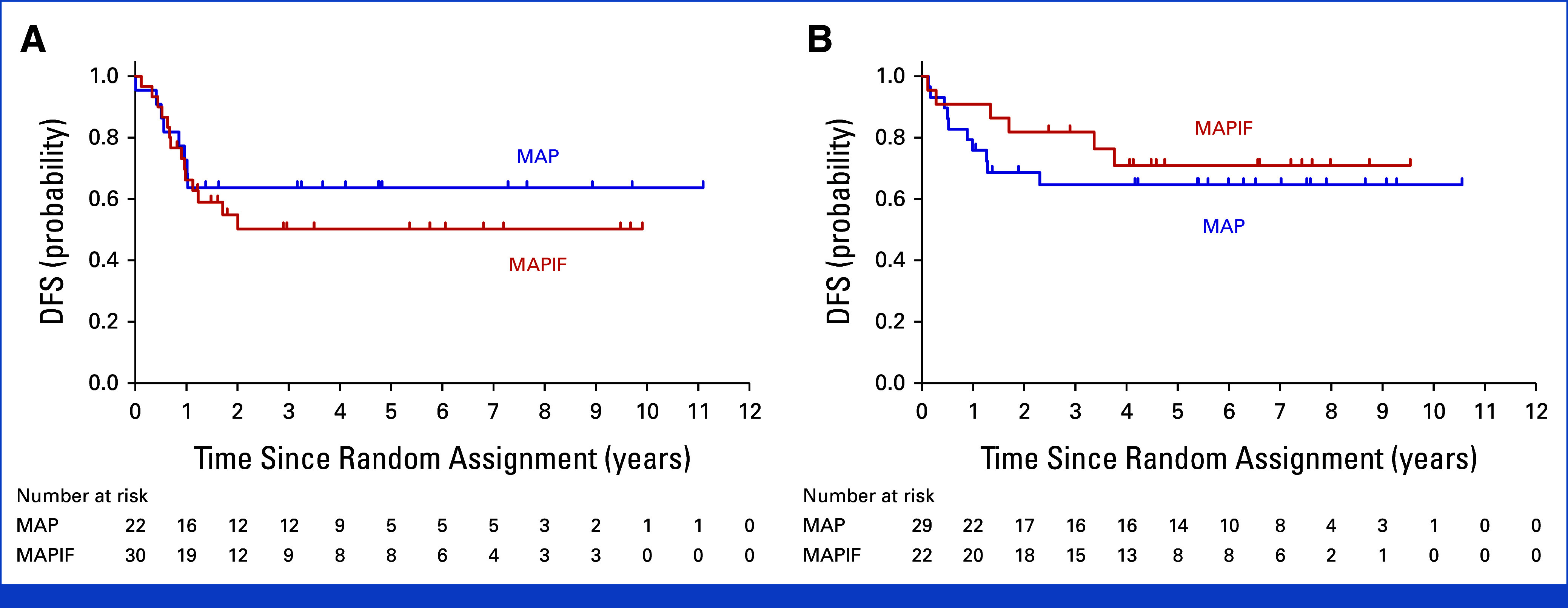
Subgroup analysis for DFS of the MAP and MAPIF arms. Kaplan-Meier curves showing DFS of patients with a histologic effect of (A) grade 1 and (B) grade 2 for the MAP and MAPIF arms. DFS, disease-free survival; MAP, methotrexate, doxorubicin, and cisplatin; MAPIF, MAP + ifosfamide.

The MAPIF arm had a higher proportion of grade 1 histologic responses than the MAP arm (58% *v* 43%), suggesting slightly worse preoperative responses in the MAPIF arm. The subgroup analysis of histologic effects showed that among the 52 patients with grade 1, the DFS in the MAP arm was longer than that in the MAPIF arm, whereas among the 51 patients with grade 2, the DFS in the MAPIF arm was longer than that in the MAP arm (Appendix Fig A2). However, the HRs from the Cox regression model, adjusted for histologic response (grade 1 *v* grade 2), were 1.030 (95% CI, 0.543 to 1.953) for DFS and 1.396 (95% CI, 0.639 to 3.050) for OS. Therefore, the impact of this imbalance on the primary result may be negligible.

Our trial findings support the EURAMOS-1 results and demonstrate its broader applicability, particularly in Asia. However, as a Japan-specific trial, limitations included small sample size, a one-sided α of .1, and a statistical power of <70%. These parameters were chosen mainly because of feasibility constraints, particularly the accrual challenges of studying this rare tumor type. Consequently, the originally planned registration period was extended from 6 years to 10.5 years. The second registration was based on histologic response evaluations at each institution. A central pathologic review was planned; however, sample shipments from each institution were delayed, resulting in only 58 (32.8%) of 177 patients in the second registration completing the review.

The reasons why IF, an active agent against osteosarcoma, fails as an effective rescue treatment remain elusive. Clinically, IF has been reported to effectively treat relapses after MAP therapy.^21,23^ However, in randomized comparative trials, including this study, adding IF did not show superiority. This finding suggests that MAP and IF targets largely overlap. Osteosarcoma cells overexpressing mutated GPC3 exhibit multidrug resistance,^26^ indicating the need for novel agents with new mechanisms of action. Furthermore, a biomarker to estimate preoperative chemotherapy effects earlier than histologic response evaluation is essential for improving treatment.^27^

Adjuvant MAPIF showed no DFS improvement over MAP, and OS tended to be lower. As expected, adjuvant MAPIF was more toxic. The JCOG0905 findings do not support adding IF in patients with PrRsps, even at a 1.5-fold higher dose than that used in EURAMOS-1. Overcoming the long-standing constraints of modifying postoperative chemotherapy on the basis of histologic response evaluation is necessary. Investigating new strategies, including circulating tumor DNA analysis and drugs with novel mechanisms of action, is crucial.

## Data Availability

A data sharing statement provided by the authors is available with this article at DOI https://doi.org/10.1200/JCO-24-01281. Individual participant data supporting the results reported in this article will not be shared because the follow-up of the patients is continued until January 2031. After January 2031, deidentified individual participant data will be shared upon approval of proposed data use by investigators from the Bone and Soft Tissue Tumor Study Group of Japan Clinical Oncology Group. Proposals should be directed to hhiraga@nho-hcc.jp. The data will be available for achieving the aims outlined in the approved proposal.

## APPENDIX

**TABLE A1.** Histologic Response Criteria

Histologic Response	Grade	Criteria
Poor responder	Grade 1	Viable tumor cell exceeds 50%
Poor responder	Grade 2	Viable tumor cell >10% and not >50%
Good responder	Grade 3	Viable tumor cell not more than 10%
Good responder	Grade 4	No viable tumor cell

NOTE. If any finding of nuclear pyknosis, karyorrhexis, or karyolysis is observed in the nucleus, it is judged as a nonviable cell. It is considered a viable tumor cell if the cytoplasm is eosinophilic, vacuolar degeneration occurs, or nuclear swelling is observed. During mapping, for areas where chemotherapy is associated with extremely reduced cellularity and viable tumor cells are mottled, the area is multiplied by 1/10 or 1/20, as appropriate, in addition to considering the remaining area of the other viable tumor cell.

**TABLE A2.** Adverse Events in Preoperative Chemotherapy

Toxicity	Grade 1, No.	Grade 2, No.	Grade 3, No.	Grade 4, No.	Grade 3-4, %	Grade 4, %	Grade 5, No.
Toxicities routinely observed in adjuvant chemotherapy							
Leukocytes	1	59	149	53	75.7	19.9	0
Hemoglobin	56	122	75	14	33.3	5.2	0
Platelets	102	61	72	21	34.8	7.9	0
Neutrophils	2	12	31	219	93.6	82	0
Nonhematologic event		17	138	110	92.9	41.2	0
Nonhematologic event, except for AST or ALT		84	127	6	49.8	2.2	0
Bilirubin	63	21	3	0	1.1	0	0
AST	23	49	128	64	71.9	24	0
ALT	10	26	129	99	85.4	37.1	0
Creatinine	51	15	3	2	1.9	0.7	0
Hypernatremia	17	0	0	0	0	0	0
Hyponatremia	223		41	1	15.7	0.4	0
Hyperkalemia	55	2	3	0	1.1	0	0
Hypokalemia	186		38	2	15	0.7	0
Hypercalcemia	58	0	1	1	0.8	0.4	0
Hypocalcemia	169	23	3	0	1.1	0	0
Hypomagnesemia	149	11	1	0	0.5	0	0
Hearing	18	16	1	0	0.4	0	0
Left ventricular diastolic dysfunction	1	0	0	0	0	0	0
Left ventricular systolic dysfunction	1	0	0	0	0	0	0
Fever	41	15	4	0	1.5	0	0
Anorexia	117	92	30	0	11.2	0	0
Vomiting	89	67	18	0	6.7	0	0
Diarrhea	29	9	4	0	1.5	0	0
Hemorrhage, GU bladder	3	1	0	0	0	0	0
Febrile neutropenia			55	0	20.6	0	0
Confusion	0	1	0	0	0	0	0
Somnolence		9	1	0	0.4	0	0
Psychosis	0	0	0	0	0	0	0
Seizure		2	0	0	0	0	0
Encephalopathy		0	0	0	0	0	0
Involuntary movement	0	0	0	0	0	0	0
Neuropathy—motor	1	1	1	0	0.4	0	0
Neuropathy—sensory	7	3	0	0	0	0	0
Dizziness	6	1	0	0	0	0	0
Cystitis	3	1	1	0	0.4	0	0
Arrhythmia—supraventricular, ventricular	3	0	0	0	0	0	0
Mucositis—oral cavity, anus	54	28	8	0	3	0	0
Infection with grade 3 or 4 ANC^a^	0	0	0	1	0.4	0.4	0
Infection with grade 0-2 ANC^a^	0	2	1	0	0.4	0	0
Mood alteration—select: agitation, depression	4	3	1	0	0.4	0	0
Toxicities not routinely observed in adjuvant chemotherapy							
Allergic reaction			2^b^				
Infection with grade 3 or 4 ANC			1				1
Infection with grade 0-2 ANC			5^c^				
Infection with unknown ANC			1				
Hypoxemia			1				
Amylase			1				
CPK			1				
Perforation, GI ileum			1				
Mucositis—esophagus, larynx, rectum			2				
Pneumothorax			1				

NOTE. The proportion of each adverse event is calculated on the basis of the number of patients reporting each type of toxicity.

Abbreviations: ANC, absolute neutrophil count; CPK, creatine phosphorus kinase; GU, genitourinary.

^a^ Infection of the bronchus and lung.

^b^ Allergic reaction after administration of methotrexate.

^c^ Five central venous catheter infections.
